# 9-[3-(Dimethyl­amino)­prop­yl]-2-trifluoro­meth­yl-9*H*-thioxanthen-9-ol

**DOI:** 10.1107/S1600536811025311

**Published:** 2011-07-02

**Authors:** Jerry P. Jasinski, James A. Golen, M. S. Siddegowda, H. S. Yathirajan, M. T. Swamy

**Affiliations:** a Department of Chemistry, Keene State College, 229 Main Street, Keene, NH 03435-2001, USA; b Department of Studies in Chemistry, University of Mysore, Manasagangotri, Mysore 570 006, India; c Department of Chemistry, Sambhram Institute of Technology, Bangalore 560 097, India

## Abstract

In the title compound, C_19_H_20_F_3_NOS, the dihedral angle between the mean planes of the two benzene rings attached to the thioxanthene ring is 41.8 (7)°; the latter has a slightly distorted boat conformation. The F atoms are disordered over three sets of sites [occupancy ratio = 0.564 (10):0.287 (10):0.148 (5)] and the methyl groups are disordered over two sets of sites [occupancy ratio = 0.72 (4):0.28 (4)]. The crystal packing is stabilized by O—H⋯N and C—H⋯S hydrogen bonds and weak C—H⋯*Cg* inter­actions.

## Related literature

For photo-initiators with excellent capabilities in UV-curing materials, see: Fouassier *et al.* (1995 ▶); Roffey (1997 ▶). For related structures, see: Post *et al.* (1975*a* ▶,*b* ▶); Liu, (2009 ▶). For puckering parameters, see: Cremer & Pople (1975 ▶).
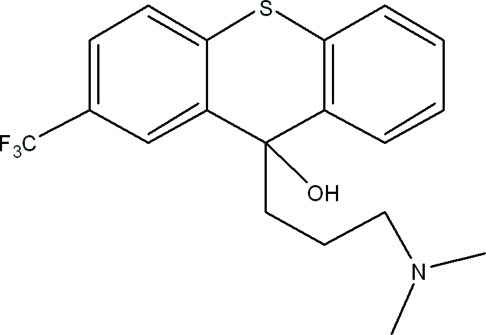


## Experimental

### 

#### Crystal data

C_19_H_20_F_3_NOS
*M* *_r_* = 367.42
Monoclinic, 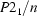

*a* = 7.6183 (3) Å
*b* = 13.9605 (4) Å
*c* = 17.4172 (7) Å
β = 101.053 (4)°
*V* = 1818.05 (11) Å^3^
*Z* = 4
Mo *K*α radiation
μ = 0.21 mm^−1^
*T* = 170 K
0.35 × 0.33 × 0.30 mm

#### Data collection

Oxford Diffraction Xcalibur Eos Gemini diffractometer
Absorption correction: multi-scan (*CrysAlis RED*; Oxford Diffraction, 2010 ▶) *T*_min_ = 0.929, *T*_max_ = 0.939
17330 measured reflections
4697 independent reflections
3901 reflections with *I* > 2σ(*I*)
*R*_int_ = 0.022

#### Refinement

*R*[*F* ^2^ > 2σ(*F* ^2^)] = 0.043
*wR*(*F* ^2^) = 0.116
*S* = 1.05
4697 reflections
306 parameters
238 restraints
H atoms treated by a mixture of independent and constrained refinement
Δρ_max_ = 0.28 e Å^−3^
Δρ_min_ = −0.29 e Å^−3^

### 

Data collection: *CrysAlis PRO* (Oxford Diffraction, 2010 ▶); cell refinement: *CrysAlis PRO*; data reduction: *CrysAlis RED* (Oxford Diffraction, 2010 ▶); program(s) used to solve structure: *SHELXS97* (Sheldrick, 2008 ▶); program(s) used to refine structure: *SHELXL97* (Sheldrick, 2008 ▶); molecular graphics: *SHELXTL* (Sheldrick, 2008 ▶); software used to prepare material for publication: *SHELXTL*.

## Supplementary Material

Crystal structure: contains datablock(s) global, I. DOI: 10.1107/S1600536811025311/tk2761sup1.cif

Structure factors: contains datablock(s) I. DOI: 10.1107/S1600536811025311/tk2761Isup2.hkl

Supplementary material file. DOI: 10.1107/S1600536811025311/tk2761Isup3.cml

Additional supplementary materials:  crystallographic information; 3D view; checkCIF report

## Figures and Tables

**Table 1:** Hydrogen-bond geometry (Å, °) *Cg*2 and *Cg*3 are the centroids of the C2–C7 and C8–C13 rings, respectively.

*D*—H⋯*A*	*D*—H	H⋯*A*	*D*⋯*A*	*D*—H⋯*A*
O1—H1*A*⋯N1	0.87 (2)	1.84 (2)	2.7141 (17)	176 (2)
C15—H15*A*⋯S1	0.99	2.76	3.4165 (15)	124
C5—H5*A*⋯*Cg*3^i^	0.95	2.96	3.798 (3)	148
C17—H17*A*⋯*Cg*2^ii^	0.99	2.97	3.949 (3)	170
C17—H17*B*⋯*Cg*3^ii^	0.99	2.83	3.659 (3)	142

Symmetry codes: (i) 
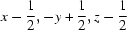
; (ii) 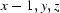.

**Fig. 1.**
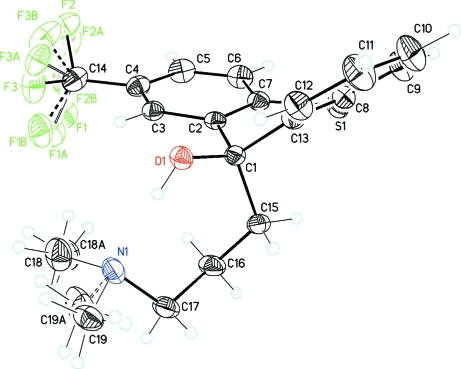
Molecular structure of (I) showing the atom labeling scheme and 50% probability displacement ellipsoids.

**Fig. 2.**
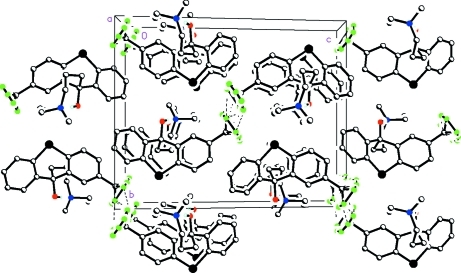
Packing diagram of the title compound viewed down the a axis. Dashed lines represent disordered C—F atoms.

## supplementary crystallographic information

### Comment

The title compound is a flupenthixol impurity with systematic IUPAC name: (*RS*)-9-[3-(dimethylamino)propyl]-2-(trifluoromethyl)-9H-thioxanthen-9-ol. Flupenthixol is a thioxanthene derivative that may exist in two isomeric forms, α and β. Flupenthixol contains 45-55 % α-flupenthixol. The pharmacological effects of flupenthixol, α- and β-flupenthixol have been compared with those of clopenthixol, chlorprothixene, fluphenazine, perphenazine, chlorpromazine and haloperidol. In most pharmacological screening tests α-flupenthixol was equipotent with fluphenazine. β-Flupenthixol showed very low pharmacological activity. As expected the potency of flupenthixol was about one half that of α-flupenthixol. Thioxanthone derivatives are good photoinitiators with excellent capabilities in UV-curing materials (Fouassier *et al.*, 1995; Roffey, 1997). The crystal structures of α-flupenthixol (Post *et al.*, 1975*b*), β-flupenthixol (Post *et al.*, 1975*a*) and 2,4-diethylthioxanthen-9-one (Liu, 2009) have been reported. In view of the importance of the title compound the crystal structure is herein reported.

In the title compound, (I), the dihedral angle between the mean planes of the two benzene rings in the thioxanthene ring is 41.8 (7) ° (Fig. 1). The thioxanthene ring is in a slightly distorted boat conformation (Cremer & Pople, 1975) with puckering parameters Q, θ, and φ = 0.591 (2) Å, 92.72 (19) ° and 359.8 (2) °, respectively). Crystal packing is stabilized by O1—H1A···N1, C15—H15A···S1 hydrogen bonds and weak C—H···Cg intermolecular interactions (Fig. 2, Table 1).

### Experimental

The title compound was obtained as a gift sample from R. L. Fine Chem. Ltd., Bangalore, India. The compound was recrystallized from dichloromethane (*M*.pt.: 389–391 K).

### Refinement

The fluorine atoms on C14 are disordered over three positions [occupancy ratio 0.564 (10); 0.287 (10); 0.148 (5)] and the methyl groups on N1 are disordered over two positions [occupancy ratio 0.72 (4); 0.28 (4)]. The O–H hydrogen atom was located by Fourier analysis and refined isotropically. All of the remaining H atoms were placed in their calculated positions and then refined using the riding model with atom–H lengths of 0.95 Å (CH), 0.99 Å (CH_2_) or 0.98 Å (CH_3_). Isotropic displacement parameters for these atoms were set to 1.18-1.20 (CH) or 1.20 (CH_2_) times *U*_eq_ of the parent atom.

### Crystal data

**Table:**

C_19_H_20_F_3_NOS	*F*(000) = 768
*M**_r_* = 367.42	*D*_x_ = 1.342 Mg m^−^^3^
Monoclinic, *P*2_1_/*n*	Mo *K*α radiation, λ = 0.71073 Å
Hall symbol: -P 2yn	Cell parameters from 9234 reflections
*a* = 7.6183 (3) Å	θ = 3.3–32.3°
*b* = 13.9605 (4) Å	µ = 0.21 mm^−^^1^
*c* = 17.4172 (7) Å	*T* = 170 K
β = 101.053 (4)°	Block, pale yellow
*V* = 1818.05 (11) Å^3^	0.35 × 0.33 × 0.30 mm
*Z* = 4	

### Data collection

**Table:**

Oxford Diffraction Xcalibur Eos Gemini diffractometer	4697 independent reflections
Radiation source: Enhance (Mo) X-ray Source	3901 reflections with *I* > 2σ(*I*)
graphite	*R*_int_ = 0.022
Detector resolution: 16.1500 pixels mm^-1^	θ_max_ = 28.7°, θ_min_ = 3.5°
ω scans	*h* = −10→10
Absorption correction: multi-scan (*CrysAlis RED*; Oxford Diffraction, 2010)	*k* = −18→15
*T*_min_ = 0.929, *T*_max_ = 0.939	*l* = −23→23
17330 measured reflections	

### Refinement

**Table:**

Refinement on *F*^2^	Primary atom site location: structure-invariant direct methods
Least-squares matrix: full	Secondary atom site location: difference Fourier map
*R*[*F*^2^ > 2σ(*F*^2^)] = 0.043	Hydrogen site location: inferred from neighbouring sites
*wR*(*F*^2^) = 0.116	H atoms treated by a mixture of independent and constrained refinement
*S* = 1.05	*w* = 1/[σ^2^(*F*_o_^2^) + (0.0506*P*)^2^ + 0.6042*P*] where *P* = (*F*_o_^2^ + 2*F*_c_^2^)/3
4697 reflections	(Δ/σ)_max_ = 0.037
306 parameters	Δρ_max_ = 0.28 e Å^−^^3^
238 restraints	Δρ_min_ = −0.29 e Å^−^^3^

### Special details

**Table:**

Geometry. All esds (except the esd in the dihedral angle between two l.s. planes) are estimated using the full covariance matrix. The cell esds are taken into account individually in the estimation of esds in distances, angles and torsion angles; correlations between esds in cell parameters are only used when they are defined by crystal symmetry. An approximate (isotropic) treatment of cell esds is used for estimating esds involving l.s. planes.
Refinement. Refinement of *F*^2^ against ALL reflections. The weighted *R*-factor *wR* and goodness of fit *S* are based on *F*^2^, conventional *R*-factors *R* are based on *F*, with *F* set to zero for negative *F*^2^. The threshold expression of *F*^2^ > σ(*F*^2^) is used only for calculating *R*-factors(gt) *etc*. and is not relevant to the choice of reflections for refinement. *R*-factors based on *F*^2^ are statistically about twice as large as those based on *F*, and *R*- factors based on ALL data will be even larger.

### Fractional atomic coordinates and isotropic or equivalent isotropic displacement parameters (Å^2^)

**Table:**

	*x*	*y*	*z*	*U*_iso_*/*U*_eq_	Occ. (<1)
S1	0.74858 (6)	0.83313 (3)	0.17618 (2)	0.04558 (12)	
F1	0.6729 (8)	0.6443 (4)	0.5165 (2)	0.0783 (14)	0.564 (10)
F2	0.9343 (6)	0.5995 (5)	0.5119 (4)	0.0815 (15)	0.564 (10)
F3	0.7169 (10)	0.5137 (4)	0.4572 (4)	0.0765 (16)	0.564 (10)
F1A	0.6033 (13)	0.6017 (10)	0.4888 (7)	0.078 (2)	0.287 (10)
F2A	0.8891 (19)	0.6238 (7)	0.5244 (5)	0.072 (3)	0.287 (10)
F3A	0.7863 (16)	0.5067 (7)	0.4533 (7)	0.059 (2)	0.287 (10)
F1B	0.5941 (16)	0.5562 (12)	0.4648 (6)	0.070 (3)	0.148 (5)
F2B	0.785 (3)	0.6465 (10)	0.5293 (6)	0.075 (3)	0.148 (5)
F3B	0.864 (2)	0.5254 (12)	0.4705 (10)	0.091 (4)	0.148 (5)
O1	0.50685 (14)	0.54112 (7)	0.18448 (6)	0.0369 (2)	
H1A	0.408 (3)	0.5332 (16)	0.2017 (13)	0.070 (6)*	
N1	0.19359 (19)	0.52532 (10)	0.23591 (10)	0.0543 (4)	
C1	0.54324 (17)	0.63994 (9)	0.17934 (8)	0.0316 (3)	
C2	0.65553 (17)	0.67633 (9)	0.25587 (8)	0.0319 (3)	
C3	0.66939 (18)	0.62297 (10)	0.32396 (8)	0.0346 (3)	
H3A	0.6162	0.5612	0.3220	0.041*	
C4	0.7599 (2)	0.65865 (11)	0.39484 (9)	0.0410 (3)	
C5	0.8404 (2)	0.74815 (12)	0.39867 (10)	0.0501 (4)	
H5A	0.8989	0.7732	0.4476	0.060*	
C6	0.8350 (2)	0.80039 (12)	0.33140 (10)	0.0473 (4)	
H6A	0.8935	0.8607	0.3334	0.057*	
C7	0.74356 (18)	0.76466 (10)	0.26013 (9)	0.0363 (3)	
C8	0.7353 (2)	0.74071 (11)	0.10656 (9)	0.0406 (3)	
C9	0.8206 (3)	0.75615 (14)	0.04376 (11)	0.0562 (4)	
H9A	0.8784	0.8155	0.0388	0.067*	
C10	0.8210 (3)	0.68539 (17)	−0.01102 (12)	0.0683 (6)	
H10A	0.8778	0.6962	−0.0543	0.082*	
C11	0.7391 (3)	0.59858 (16)	−0.00345 (11)	0.0641 (5)	
H11A	0.7412	0.5494	−0.0410	0.077*	
C12	0.6541 (2)	0.58328 (13)	0.05899 (9)	0.0478 (4)	
H12A	0.5992	0.5231	0.0642	0.057*	
C13	0.64777 (18)	0.65429 (10)	0.11405 (8)	0.0363 (3)	
C14	0.7655 (3)	0.60126 (14)	0.46712 (10)	0.0534 (4)	
C15	0.36815 (18)	0.69875 (11)	0.15859 (9)	0.0411 (3)	
H15A	0.4009	0.7658	0.1494	0.049*	
H15B	0.2994	0.6740	0.1085	0.049*	
C16	0.2439 (2)	0.69987 (11)	0.21787 (11)	0.0489 (4)	
H16A	0.3181	0.6992	0.2712	0.059*	
H16B	0.1758	0.7607	0.2119	0.059*	
C17	0.1118 (2)	0.61725 (12)	0.21095 (12)	0.0525 (4)	
H17A	0.0208	0.6320	0.2429	0.063*	
H17B	0.0494	0.6119	0.1558	0.063*	
C18	0.2274 (12)	0.5222 (12)	0.3225 (4)	0.095 (2)	0.72 (4)
H18A	0.3144	0.5718	0.3435	0.142*	0.72 (4)
H18B	0.2749	0.4591	0.3404	0.142*	0.72 (4)
H18C	0.1153	0.5336	0.3407	0.142*	0.72 (4)
C19	0.0843 (17)	0.4459 (7)	0.2028 (8)	0.100 (3)	0.72 (4)
H19A	0.0712	0.4470	0.1457	0.150*	0.72 (4)
H19B	−0.0339	0.4509	0.2169	0.150*	0.72 (4)
H19C	0.1412	0.3857	0.2232	0.150*	0.72 (4)
C18A	0.224 (3)	0.5017 (14)	0.3172 (8)	0.067 (4)	0.28 (4)
H18D	0.2296	0.4320	0.3234	0.101*	0.28 (4)
H18E	0.1268	0.5272	0.3406	0.101*	0.28 (4)
H18F	0.3380	0.5298	0.3435	0.101*	0.28 (4)
C19A	0.061 (2)	0.4530 (13)	0.1931 (10)	0.051 (4)	0.28 (4)
H19D	0.0576	0.4581	0.1367	0.076*	0.28 (4)
H19E	−0.0578	0.4659	0.2041	0.076*	0.28 (4)
H19F	0.0989	0.3882	0.2108	0.076*	0.28 (4)

### Atomic displacement parameters (Å^2^)

**Table:**

	*U*^11^	*U*^22^	*U*^33^	*U*^12^	*U*^13^	*U*^23^
S1	0.0509 (2)	0.03275 (19)	0.0553 (2)	−0.00550 (15)	0.01586 (18)	0.00201 (15)
F1	0.089 (3)	0.098 (3)	0.055 (2)	0.031 (2)	0.032 (2)	0.0041 (17)
F2	0.0630 (17)	0.098 (4)	0.072 (3)	0.0147 (17)	−0.0148 (16)	0.020 (2)
F3	0.112 (4)	0.062 (2)	0.0513 (16)	−0.017 (3)	0.005 (3)	0.0106 (15)
F1A	0.084 (4)	0.102 (6)	0.061 (5)	0.024 (4)	0.041 (3)	0.013 (4)
F2A	0.098 (7)	0.070 (4)	0.034 (3)	0.003 (5)	−0.020 (4)	−0.012 (2)
F3A	0.081 (5)	0.045 (2)	0.045 (3)	0.007 (3)	−0.004 (4)	−0.0009 (19)
F1B	0.092 (5)	0.079 (7)	0.040 (5)	−0.015 (5)	0.015 (4)	0.007 (4)
F2B	0.090 (7)	0.093 (6)	0.040 (4)	−0.004 (6)	0.009 (6)	−0.006 (4)
F3B	0.095 (7)	0.088 (7)	0.083 (7)	0.036 (6)	−0.005 (6)	0.020 (5)
O1	0.0366 (5)	0.0292 (5)	0.0457 (6)	−0.0035 (4)	0.0102 (4)	−0.0017 (4)
N1	0.0462 (8)	0.0456 (7)	0.0770 (10)	−0.0004 (6)	0.0265 (7)	0.0158 (7)
C1	0.0277 (6)	0.0294 (6)	0.0373 (7)	−0.0005 (5)	0.0057 (5)	0.0019 (5)
C2	0.0257 (6)	0.0314 (6)	0.0391 (7)	0.0030 (5)	0.0071 (5)	−0.0029 (5)
C3	0.0318 (6)	0.0339 (6)	0.0383 (7)	0.0031 (5)	0.0077 (5)	−0.0024 (5)
C4	0.0403 (7)	0.0446 (8)	0.0378 (7)	0.0080 (6)	0.0063 (6)	−0.0045 (6)
C5	0.0520 (9)	0.0505 (9)	0.0451 (9)	−0.0004 (7)	0.0027 (7)	−0.0155 (7)
C6	0.0462 (8)	0.0374 (7)	0.0575 (10)	−0.0054 (6)	0.0083 (7)	−0.0130 (7)
C7	0.0323 (6)	0.0316 (6)	0.0458 (8)	0.0015 (5)	0.0097 (6)	−0.0028 (5)
C8	0.0369 (7)	0.0427 (8)	0.0428 (8)	−0.0026 (6)	0.0093 (6)	0.0024 (6)
C9	0.0581 (10)	0.0584 (10)	0.0568 (10)	−0.0140 (8)	0.0227 (8)	0.0040 (8)
C10	0.0771 (13)	0.0818 (14)	0.0547 (11)	−0.0166 (11)	0.0343 (10)	−0.0042 (10)
C11	0.0743 (13)	0.0736 (13)	0.0495 (10)	−0.0156 (10)	0.0252 (9)	−0.0170 (9)
C12	0.0485 (9)	0.0528 (9)	0.0429 (8)	−0.0109 (7)	0.0111 (7)	−0.0079 (7)
C13	0.0311 (6)	0.0413 (7)	0.0357 (7)	−0.0030 (5)	0.0045 (5)	0.0006 (5)
C14	0.0584 (10)	0.0599 (10)	0.0412 (8)	0.0141 (8)	0.0076 (7)	−0.0022 (7)
C15	0.0296 (6)	0.0377 (7)	0.0538 (9)	0.0010 (5)	0.0028 (6)	0.0079 (6)
C16	0.0329 (7)	0.0379 (8)	0.0779 (12)	0.0043 (6)	0.0158 (7)	0.0000 (7)
C17	0.0317 (7)	0.0475 (9)	0.0802 (12)	0.0003 (6)	0.0158 (8)	0.0068 (8)
C18	0.072 (4)	0.133 (6)	0.089 (3)	0.010 (4)	0.037 (3)	0.057 (3)
C19	0.111 (6)	0.055 (3)	0.147 (6)	−0.028 (4)	0.055 (4)	0.011 (3)
C18A	0.077 (9)	0.056 (6)	0.063 (6)	−0.027 (6)	0.001 (6)	0.000 (5)
C19A	0.039 (5)	0.042 (6)	0.072 (7)	−0.007 (4)	0.014 (4)	−0.007 (5)

### Geometric parameters (Å, °)

**Table:**

S1—C7	1.7535 (15)	C6—H6A	0.9500
S1—C8	1.7602 (16)	C8—C9	1.392 (2)
F1—C14	1.354 (3)	C8—C13	1.397 (2)
F2—C14	1.370 (5)	C9—C10	1.374 (3)
F3—C14	1.280 (5)	C9—H9A	0.9500
F1A—C14	1.360 (7)	C10—C11	1.381 (3)
F1A—F3A	2.102 (9)	C10—H10A	0.9500
F2A—C14	1.273 (8)	C11—C12	1.385 (2)
F3A—C14	1.357 (9)	C11—H11A	0.9500
F1B—C14	1.443 (10)	C12—C13	1.386 (2)
F2B—C14	1.237 (9)	C12—H12A	0.9500
F3B—C14	1.292 (10)	C15—C16	1.529 (2)
O1—C1	1.4134 (15)	C15—H15A	0.9900
O1—H1A	0.87 (2)	C15—H15B	0.9900
N1—C18A	1.429 (14)	C16—C17	1.520 (2)
N1—C19	1.439 (8)	C16—H16A	0.9900
N1—C17	1.456 (2)	C16—H16B	0.9900
N1—C18	1.481 (7)	C17—H17A	0.9900
N1—C19A	1.517 (12)	C17—H17B	0.9900
C1—C13	1.5213 (19)	C18—H18A	0.9800
C1—C2	1.5263 (19)	C18—H18B	0.9800
C1—C15	1.5489 (19)	C18—H18C	0.9800
C2—C3	1.3873 (19)	C19—H19A	0.9800
C2—C7	1.3989 (19)	C19—H19B	0.9800
C3—C4	1.386 (2)	C19—H19C	0.9800
C3—H3A	0.9500	C18A—H18D	0.9800
C4—C5	1.388 (2)	C18A—H18E	0.9800
C4—C14	1.486 (2)	C18A—H18F	0.9800
C5—C6	1.374 (2)	C19A—H19D	0.9800
C5—H5A	0.9500	C19A—H19E	0.9800
C6—C7	1.394 (2)	C19A—H19F	0.9800
			
C7—S1—C8	99.63 (7)	F3—C14—F1A	77.7 (5)
C1—O1—H1A	109.8 (15)	F3B—C14—F1A	122.8 (9)
C18A—N1—C19	101.1 (8)	F2B—C14—F2	64.3 (9)
C18A—N1—C17	118.6 (10)	F3A—C14—F2	87.4 (5)
C19—N1—C17	112.2 (6)	F3—C14—F2	106.0 (4)
C19—N1—C18	111.1 (5)	F1—C14—F2	101.1 (3)
C17—N1—C18	108.1 (6)	F3B—C14—F2	59.2 (9)
C18A—N1—C19A	107.5 (9)	F1A—C14—F2	130.2 (5)
C17—N1—C19A	103.5 (8)	F2B—C14—F1B	101.8 (7)
C18—N1—C19A	116.8 (9)	F3A—C14—F1B	73.0 (7)
O1—C1—C13	108.24 (11)	F2A—C14—F1B	131.2 (7)
O1—C1—C2	110.70 (11)	F3—C14—F1B	48.9 (7)
C13—C1—C2	108.84 (10)	F1—C14—F1B	68.4 (7)
O1—C1—C15	111.19 (11)	F3B—C14—F1B	99.1 (8)
C13—C1—C15	107.85 (11)	F2—C14—F1B	138.9 (6)
C2—C1—C15	109.92 (11)	F2B—C14—C4	116.4 (6)
C3—C2—C7	118.09 (13)	F3A—C14—C4	111.1 (6)
C3—C2—C1	120.40 (12)	F2A—C14—C4	115.3 (6)
C7—C2—C1	121.50 (12)	F3—C14—C4	116.1 (3)
C4—C3—C2	120.78 (13)	F1—C14—C4	111.4 (2)
C4—C3—H3A	119.6	F3B—C14—C4	113.8 (7)
C2—C3—H3A	119.6	F1A—C14—C4	110.6 (3)
C3—C4—C5	120.41 (15)	F2—C14—C4	111.3 (3)
C3—C4—C14	119.30 (15)	F1B—C14—C4	109.4 (4)
C5—C4—C14	120.27 (15)	C16—C15—C1	117.70 (13)
C6—C5—C4	119.75 (15)	C16—C15—H15A	107.9
C6—C5—H5A	120.1	C1—C15—H15A	107.9
C4—C5—H5A	120.1	C16—C15—H15B	107.9
C5—C6—C7	119.83 (15)	C1—C15—H15B	107.9
C5—C6—H6A	120.1	H15A—C15—H15B	107.2
C7—C6—H6A	120.1	C17—C16—C15	115.14 (14)
C6—C7—C2	121.02 (14)	C17—C16—H16A	108.5
C6—C7—S1	117.41 (11)	C15—C16—H16A	108.5
C2—C7—S1	121.55 (11)	C17—C16—H16B	108.5
C9—C8—C13	120.68 (15)	C15—C16—H16B	108.5
C9—C8—S1	116.99 (12)	H16A—C16—H16B	107.5
C13—C8—S1	122.30 (11)	N1—C17—C16	113.97 (13)
C10—C9—C8	119.86 (17)	N1—C17—H17A	108.8
C10—C9—H9A	120.1	C16—C17—H17A	108.8
C8—C9—H9A	120.1	N1—C17—H17B	108.8
C9—C10—C11	120.25 (17)	C16—C17—H17B	108.8
C9—C10—H10A	119.9	H17A—C17—H17B	107.7
C11—C10—H10A	119.9	N1—C18—H18A	109.5
C10—C11—C12	119.87 (17)	N1—C18—H18B	109.5
C10—C11—H11A	120.1	H18A—C18—H18B	109.5
C12—C11—H11A	120.1	N1—C18—H18C	109.5
C13—C12—C11	121.09 (16)	H18A—C18—H18C	109.5
C13—C12—H12A	119.5	H18B—C18—H18C	109.5
C11—C12—H12A	119.5	N1—C19—H19A	109.5
C12—C13—C8	118.20 (14)	N1—C19—H19B	109.5
C12—C13—C1	121.02 (13)	H19A—C19—H19B	109.5
C8—C13—C1	120.74 (13)	N1—C19—H19C	109.5
F2B—C14—F3A	130.8 (9)	H19A—C19—H19C	109.5
F3A—C14—F2A	106.6 (6)	H19B—C19—H19C	109.5
F2B—C14—F3	126.1 (7)	N1—C18A—H18D	109.5
F2A—C14—F3	119.6 (6)	N1—C18A—H18E	109.5
F3A—C14—F1	129.6 (6)	H18D—C18A—H18E	109.5
F2A—C14—F1	77.8 (5)	N1—C18A—H18F	109.5
F3—C14—F1	109.7 (3)	H18D—C18A—H18F	109.5
F2B—C14—F3B	113.9 (9)	H18E—C18A—H18F	109.5
F2A—C14—F3B	80.3 (9)	N1—C19A—H19D	109.5
F3—C14—F3B	51.2 (9)	N1—C19A—H19E	109.5
F1—C14—F3B	134.7 (7)	H19D—C19A—H19E	109.5
F2B—C14—F1A	73.7 (9)	N1—C19A—H19F	109.5
F3A—C14—F1A	101.4 (6)	H19D—C19A—H19F	109.5
F2A—C14—F1A	110.8 (6)	H19E—C19A—H19F	109.5
			
O1—C1—C2—C3	−16.56 (16)	F1A—F3A—C14—F3	−10 (2)
C13—C1—C2—C3	−135.40 (12)	F1A—F3A—C14—F1	28.2 (6)
C15—C1—C2—C3	106.68 (14)	F1A—F3A—C14—F3B	141.1 (17)
O1—C1—C2—C7	164.96 (11)	F1A—F3A—C14—F2	130.6 (5)
C13—C1—C2—C7	46.11 (16)	F1A—F3A—C14—F1B	−12.8 (7)
C15—C1—C2—C7	−71.81 (15)	F1A—F3A—C14—C4	−117.6 (5)
C7—C2—C3—C4	3.40 (19)	F3A—F1A—C14—F2B	−129.4 (9)
C1—C2—C3—C4	−175.14 (12)	F3A—F1A—C14—F2A	−112.9 (7)
C2—C3—C4—C5	−0.8 (2)	F3A—F1A—C14—F3	4.3 (8)
C2—C3—C4—C14	177.44 (13)	F3A—F1A—C14—F1	−143.5 (7)
C3—C4—C5—C6	−2.0 (2)	F3A—F1A—C14—F3B	−21.1 (10)
C14—C4—C5—C6	179.71 (15)	F3A—F1A—C14—F2	−96.4 (8)
C4—C5—C6—C7	2.2 (2)	F3A—F1A—C14—F1B	24.2 (13)
C5—C6—C7—C2	0.4 (2)	F3A—F1A—C14—C4	117.9 (6)
C5—C6—C7—S1	−177.87 (13)	C3—C4—C14—F2B	−153.9 (11)
C3—C2—C7—C6	−3.2 (2)	C5—C4—C14—F2B	24.4 (11)
C1—C2—C7—C6	175.29 (13)	C3—C4—C14—F3A	39.4 (6)
C3—C2—C7—S1	175.02 (10)	C5—C4—C14—F3A	−142.3 (6)
C1—C2—C7—S1	−6.46 (17)	C3—C4—C14—F2A	160.8 (7)
C8—S1—C7—C6	149.19 (12)	C5—C4—C14—F2A	−20.9 (7)
C8—S1—C7—C2	−29.12 (12)	C3—C4—C14—F3	13.7 (5)
C7—S1—C8—C9	−148.84 (14)	C5—C4—C14—F3	−168.1 (4)
C7—S1—C8—C13	29.48 (14)	C3—C4—C14—F1	−112.9 (4)
C13—C8—C9—C10	−0.8 (3)	C5—C4—C14—F1	65.4 (4)
S1—C8—C9—C10	177.53 (17)	C3—C4—C14—F3B	70.5 (11)
C8—C9—C10—C11	−0.9 (3)	C5—C4—C14—F3B	−111.2 (11)
C9—C10—C11—C12	0.9 (4)	C3—C4—C14—F1A	−72.4 (8)
C10—C11—C12—C13	0.7 (3)	C5—C4—C14—F1A	105.9 (8)
C11—C12—C13—C8	−2.3 (3)	C3—C4—C14—F2	135.1 (4)
C11—C12—C13—C1	175.65 (16)	C5—C4—C14—F2	−46.7 (4)
C9—C8—C13—C12	2.4 (2)	C3—C4—C14—F1B	−39.2 (8)
S1—C8—C13—C12	−175.88 (12)	C5—C4—C14—F1B	139.0 (8)
C9—C8—C13—C1	−175.60 (15)	O1—C1—C15—C16	64.51 (17)
S1—C8—C13—C1	6.1 (2)	C13—C1—C15—C16	−176.96 (12)
O1—C1—C13—C12	16.06 (18)	C2—C1—C15—C16	−58.43 (16)
C2—C1—C13—C12	136.43 (14)	C1—C15—C16—C17	−86.15 (18)
C15—C1—C13—C12	−104.34 (16)	C18A—N1—C17—C16	84.4 (9)
O1—C1—C13—C8	−166.03 (13)	C19—N1—C17—C16	−158.3 (5)
C2—C1—C13—C8	−45.65 (17)	C18—N1—C17—C16	78.8 (4)
C15—C1—C13—C8	73.57 (16)	C19A—N1—C17—C16	−156.7 (7)
F1A—F3A—C14—F2B	78.2 (12)	C15—C16—C17—N1	71.3 (2)
F1A—F3A—C14—F2A	116.0 (6)		

### Hydrogen-bond geometry (Å, °)

**Table:**

Cg2 and Cg3 are the centroids of the C2–C7 and C8–C13 rings, respectively.

**Table:**

*D*—H···*A*	*D*—H	H···*A*	*D*···*A*	*D*—H···*A*
O1—H1A···N1	0.87 (2)	1.84 (2)	2.7141 (17)	176 (2)
C15—H15A···S1	0.99	2.76	3.4165 (15)	124.
C5—H5A···Cg3^i^	0.95	2.96	3.798 (3)	148
C17—H17A···Cg2^ii^	0.99	2.97	3.949 (3)	170
C17—H17B···Cg3^ii^	0.99	2.83	3.659 (3)	142

Symmetry codes: (i) *x*−1/2, −*y*+1/2, *z*−1/2; (ii) *x*−1, *y*, *z*.
